# Transcriptome analysis of the plant pathogen *Sclerotinia sclerotiorum* interaction with resistant and susceptible canola (*Brassica napus*) lines

**DOI:** 10.1371/journal.pone.0229844

**Published:** 2020-03-11

**Authors:** Kishore Chittem, William R. Yajima, Rubella S. Goswami, Luis E. del Río Mendoza

**Affiliations:** 1 Department of Plant Pathology, North Dakota State University, Fargo, North Dakota, United States of America; 2 USDA-APHIS, Riverdale, Maryland, United States of America; University of Nebraska-Lincoln, UNITED STATES

## Abstract

Sclerotinia stem rot is an economically important disease of canola (*Brassica napus*) and is caused by the fungal pathogen *Sclerotinia sclerotiorum*. This study evaluated the differential gene expression patterns of *S*. *sclerotiorum* during disease development on two canola lines differing in susceptibility to this pathogen. Sequencing of the mRNA libraries derived from inoculated petioles and mycelium grown on liquid medium generated approximately 164 million Illumina reads, including 95 million 75-bp-single reads, and 69 million 50-bp-paired end reads. Overall, 36% of the quality filter-passed reads were mapped to the *S*. *sclerotiorum* reference genome. On the susceptible line, 1301 and 1214 *S*. *sclerotiorum* genes were differentially expressed at early (8–16 hours post inoculation (hpi)) and late (24–48 hpi) infection stages, respectively, while on the resistant line, 1311 and 1335 genes were differentially expressed at these stages, respectively. Gene ontology (GO) categories associated with cell wall degradation, detoxification of host metabolites, peroxisome related activities like fatty acid ß-oxidation, glyoxylate cycle, oxidoreductase activity were significantly enriched in the up-regulated gene sets on both susceptible and resistant lines. Quantitative RT-PCR of six selected DEGs further validated the RNA-seq differential gene expression analysis. The regulation of effector genes involved in host defense suppression or evasion during the early infection stage, and the expression of effectors involved in host cell death in the late stage of infection provide supporting evidence for a two-phase infection model involving a brief biotrophic phase during early stages of infection. The findings from this study emphasize the role of peroxisome related pathways along with cell wall degradation and detoxification of host metabolites as the key mechanisms underlying pathogenesis of *S*. *sclerotiorum* on *B*. *napus*.

## Introduction

*Sclerotinia sclerotiorum* (Lib.) de Bary, is a very efficient plant pathogen that affects a wide range of crops and is capable of infecting plant tissues above or below the soil surface. Diseases caused by this pathogen are favored by cool wet conditions [1]. In canola (*Brassica napus* L.), this pathogen is primarily responsible for causing Sclerotinia stem rot (SSR), a yield-reducing disease endemic to canola-producing areas worldwide. Each percent increase in SSR incidence can reduce potential canola yield by 0.5% [2]. Diseases caused by *S*. *sclerotiorum* are currently controlled by fungicides [3, 4], biological formulations [5, 6], and quantitative genetic disease resistance [7].

Understanding the molecular mechanisms employed by the pathogen during the infection process is essential for identifying novel targets for SSR management. As common with many broad-host range pathogens, the molecular aspects of *S*. *sclerotiorum* pathogenicity generally studied have concentrated on the roles of hydrolytic cell wall-degrading enzymes (CWDEs) [1, 8, 9]. *S*. *sclerotiorum* is known to produce several pectinases, including both endo- (*Sspg1*, *Sspg3*, *Sspg5*, and *Sspg6*) and exo- (*Ssxpg1* and *Ssxpg2*) polygalacturonases [9]. Apart from polygalacturonases, gene disruption mutants of an arabinofuranosidase/β-xylosidase precursor (*Ssaxp*) and an endo-β-1, 4-xylanase (*SsXyl1*) showed either reduction or loss of virulence, indicating their importance as virulence factors [10, 11].

Oxalic acid (OA) has by far been the most studied *S*. *sclerotiorum* virulence factor to date [12, 13]. OA plays multiple roles in virulence of *S*. *sclerotiorum*, manipulating the host redox environment [13], inducing programmed cell death [14], detoxifying calcium, and mediating pH signaling [15, 16]. OA has long been considered an essential factor for pathogenicity of *S*. *sclerotiorum*. Recent studies using targeted mutants of the oxaloacetate acetylhydrolase gene (*Ssoah1*), responsible for biogenesis and accumulation of OA, showed that OA is required for virulence, but not essential for pathogenicity on all hosts [17, 18]. The creation of an acidic pH environment during host colonization rather than OA production per se has been suggested to be primary requirement for the colonization stage of pathogenesis [17].

Several studies have reported the role of secreted effector genes in pathogenicity or virulence of this pathogen. A small cysteine rich secreted cyanovirin-N-homology domain protein encoding gene *SsCVNH* [19], and a gene encoding small secreted hypothetical protein Ssv263 [20] were shown to be essential for full virulence of *S*. *sclerotiorum*. A compound appressorium formation related gene1 *SsCaf1* [21] and an Rhs repeat-containing protein encoding gene *Ss-Rhs1* [22] were shown to be required for host penetration and initial hyphal infection. Secreted effectors including an integrin-like protein SsITL [23], and a chorismate mutase SsCM1 [24] are known to suppress host resistance by interfering with jasmonic acid/ethylene signaling pathway and salicylic acid signaling pathways, respectively. Other secreted effectors, like the necrosis and ethylene-inducing peptides SsNep1 and SsNep2 [25], a small secreted virulence-related protein SsSSVP1 [26], and the cerato-platanin SsCP1 [27], are known to induce host cell death and necrosis.

Until recently, most of the reports involving *S*. *sclerotiorum* and canola, were limited to small scale EST studies [28, 29]; however, availability of the *S*. *sclerotiorum* genome sequence and the advances in sequencing technologies, particularly RNA-Seq have facilitated the study of global transcriptional changes occurring during pathogenesis [30, 31]. Transcriptome sequencing has been used to study the interaction of *S*. *sclerotiorum* with various crop hosts [19, 32–34] and recently with *B*. *napus* [35, 36] and *B*. *oleracea* [37]. In this study, for the first time we investigated the global transcriptional changes in *S*. *sclerotiorum* during infection of canola plants differing in their susceptibility to the pathogen.

In contrast to the widely accepted necrotrophic nature of the pathogen [1], a two-phase infection model involving a brief biotrophic or basic compatibility phase characterized by host resistance suppression and subverting of host defenses followed by a necrotrophic phase was proposed based on cytological and molecular and genomic evidences [38–40]. This brief initial phase appears to be partly facilitated by oxalic acid (OA) and by secreted effectors, which help *S*. *sclerotiorum* evade host recognition and suppress host defense signaling pathways [23, 24, 39]. The role of other effectors in avoiding host recognition and/or suppressing host defense responses is yet to be determined. We initiated this transcriptome sequencing study to gain better understanding of the molecular mechanisms underlying the interaction of *S*. *sclerotiorum* and canola and it provided insights into the temporal aspects of important mechanisms/pathways employed by *S*. *sclerotiorum* for successful infection of canola.

## Materials and methods

### Plant material and fungal strain

Two *B*. *napus* doubled haploid lines differing in their susceptibility to *S*. *Sclerotiorum* were used in this study. Both lines, NEP32 (susceptible) and NEP63 (resistant) were developed via microspore culture and were evaluated for their reaction to SSR [41]. The susceptible line NEP32 was derived from spring type canola variety Helga (PI649136), and the resistant line NEP63 was developed from a cross between Helga and winter type canola accession PI458940. When inoculated using the petiole inoculation technique [42], the susceptible line develops lesions on the stem that expand to >4 cm in length in length with 100% girdling within 8 days post inoculation, eventually leading to wilting and death. Within the same period, the average lesion size on the resistant line is limited to <1cm in length and <40% stem girdling, typically surrounded by purple margins suggesting accumulation of anthocyanins. The highly aggressive strain 1980 of *S*. *sclerotiorum* was used for inoculations and culture controls. Strain 1980 was chosen for this study as it is the genome sequenced strain [30, 31].

### Plant growth and inoculation

The seeds of NEP32 and NEP63 were surface sterilized by soaking them in 3% NaOCl for one minute followed by immersion in 70% EtOH for one minute and were rinsed three times in sterilized deionized water for 1 minute. Seeds were planted in SunGro Sunshine mix #1 (Sun Gro Horticulture, MA) in 4 x 10 plastic plots with one seed per pot and plants were grown for 4 weeks in a growth chamber with a 16 h photoperiod, 21° C/16° C day/night temperature, and 60% relative humidity. Once germinated, seedlings were watered as necessary and fertilized once a week with 20-20-20 fertilizer. Four weeks after planting, plants were infected following an established petiole inoculation technique [42]. Briefly, the petiole of one leaf per plant was cut approximately 2.5 cm away from the stem and the stump was capped with PDA plugs containing hyphal tips of an actively growing 2 days-old *S*. *sclerotiorum* colony using 1 mL pipette tip. Inoculated petioles (≈ 2.5cm) were harvested at 8, 16, 24, and 48 hours post inoculation (hpi), flash frozen in liquid nitrogen and stored at -80° C until RNA extraction. Each treatment—time point consisted of three biological replicates; petioles collected from 5 individual plants were pooled to constitute one biological replicate.

### RNA extraction, library preparation and sequencing

Total RNA was extracted from inoculated petioles using RNeasy mini kit, and mRNA was isolated using Oligotex mRNA mini kit (Qiagen Inc. Valencia, CA) following manufacturers’ instructions. cDNA libraries were constructed using NEBNext mRNA sample prep kit and NEBNext Multiplex Oligos kit (New England Biolabs, Ipswich, MA). Similar procedure was followed to construct cDNA libraries from *S*. *sclerotiorum* mycelia grown on PDB in 10cm petri plates for 48 h under the same conditions described above. For sequencing, RNA from 8 and 16 hpi were pooled and will be referred to as early infection stage (T1) henceforth. Similarly, RNA from 24 and 48 hpi were pooled and are referred to as late infection stage (T2). mRNA libraries were sequenced at the University of Minnesota Biomedical Genomics Center. Of the three biological replicates, two replicates per each treatment-time point were sequenced on Illumina GA-IIx platform (1X 76 bp Single Reads), while one replicate was sequenced on Illumina HiSeq 2500 platform (2X 50 bp Paired End).

### Quality control and read mapping to the reference genome

Quality checks for the raw fastq files were conducted through a pipeline consisting of FastQC [43] and FastX toolkit (http://hannonlab.cshl.edu/fastx_toolkit/) to retain reads that were at least 50 bp long with a minimum quality score of 30. Reference genomes and the corresponding annotations for *S*. *sclerotiorum* and *B*. *napus* were downloaded from ensemble fungi (ftp://ftp.ensemblgenomes.org/pub/release-42/fungi/) and genoscope (http://www.genoscope.cns.fr/brassicanapus/data/), respectively. The most recent full genome sequence of *S*. *sclerotiorum* (Sclerotinia sclerotiorum_1980_uf_70_gca_001857865) [31] was used. Indexes for each reference genome were built and quality filter-passed reads were mapped to the reference genomes using HiSat2 [44]. Raw read counts mapped to each gene from the HiSat2 generated alignments were obtained using the featureCounts command [45] of the Subread package [46].

### Differential gene expression analysis

Differential gene expression (DGE) analysis was conducted using edgeR package [47]. The raw count data were normalized with trimmed mean of means (TMM) normalization method implemented in edgeR [48]. Principal component analysis (PCA) was conducted to determine relatedness of the biological replicates. PCA plots were generated using scatterplot3d [49] package in R. Statistical analysis was performed using negative binomial distribution extended to generalized linear models [50]. Pairwise contrasts were performed following quasi-likelihood F tests [51]. A false discovery rate (FDR) cutoff of 0.05 was applied to account for multiple testing correction. A gene was considered as differentially expressed when the change in expression level was ≥ 2-fold (absolute value of log 2fold change (l2fc) ≥ 1), with an FDR-adjusted p-value < 0.05.

### Functional classification and enrichment analyses of DEGs

Gene ontology (GO) enrichment analysis was performed in Blast2GO [52]. GO terms were assigned to *S*. *sclerotiorum* total genes list in Blast2GO, which were used as the background list for enrichment analysis. Fishers’ exact test implemented in Blast2GO was used to identify significantly enriched GO categories. A GO category was considered significantly enriched only when the p-value for that category was < 0.05 after applying FDR correction.

### Differential gene expression data validation

The RNA-seq differential gene expression data was validated by performing qRT-PCR on six selected genes. Primers for qRT-PCR were designed using Primers-Blast [53]. QuantiTect reverse transcription kit (Qiagen Inc. Valencia, CA) was used to synthesize cDNA from total RNA. Real-time quantification was performed using two technical replications in a BioRad CFX96 Real-Time system using iTaq Universal SYBR Green Supermix with the following cycling conditions: 95° C for 30 s followed by 40 cycles of 95° C for 10 s and 60° C for 30 s. Expression levels of the DEGs were normalized against the *S*. *sclerotiorum* actin gene (sscle_14g099090) and the relative expression levels were calculated using the 2^-ΔΔCt^ method.

## Results

### Disease development

**Fig 1:**
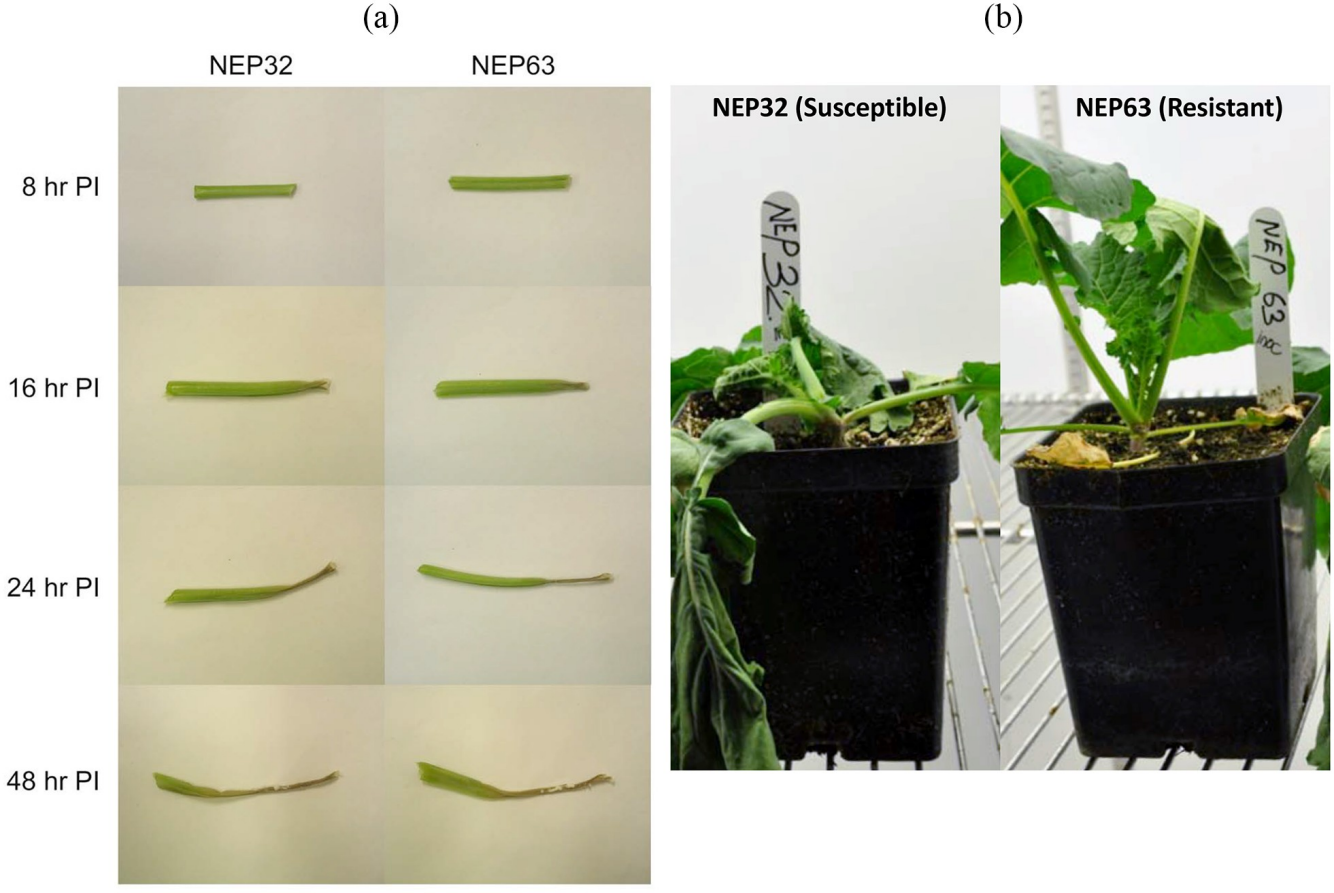
Disease development on resistant and susceptible canola lines. (a). Comparison of the appearance of representative inoculated petioles from both canola lines. The necrotic lesion spreads from the point of inoculation (i.e., petiole tip) along the length of the petiole. There are no apparent differences between NEP32 and NEP63 canola petioles. (b). Appearance of resistant (NEP 63) and susceptible (NEP32) canola four days post inoculation with *S*. *sclerotiorum*. Disease symptoms are more apparent in the susceptible line.

There were no observable phenotypic differences between the susceptible and resistant canola lines at all sampling time points (Fig 1a). In both lines, necrotic lesions first appeared at point of inoculation (petiole tip) at 16 hpi and gradually spread along the length of the petiole by 48 hpi. Non-inoculated controls never showed necrotic lesion. In the susceptible line, by 72 hpi the lesions extended into stem. In the resistant line, the lesion growth was arrested at the nodes, surrounded by purple margin and did not extend into the stem. The differences in symptoms between the susceptible and resistant canola lines were clearly apparent four days post inoculation (Fig 1b).

### Sequencing and mapping

**Fig 2:**
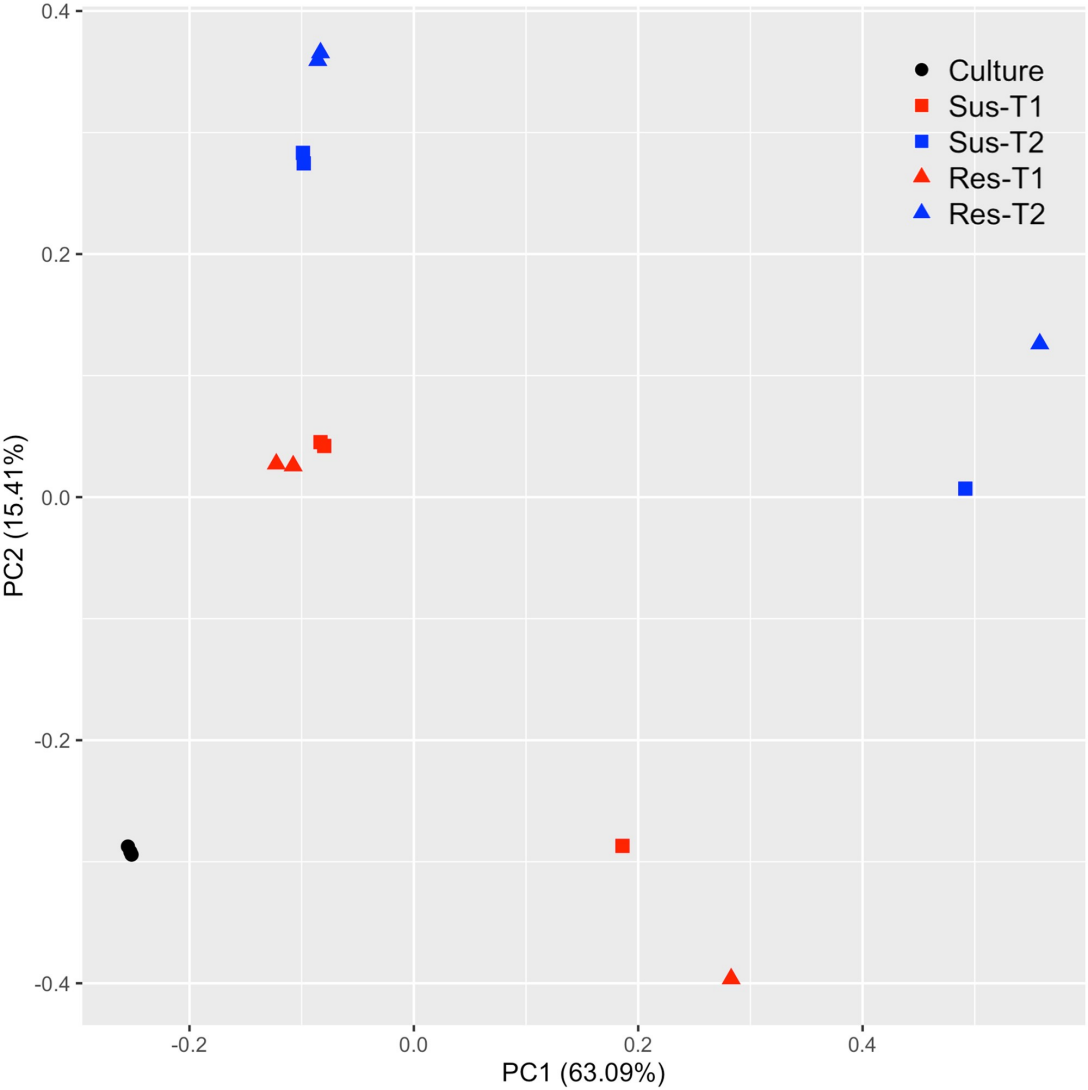
Principal component analysis of transcriptome expression. The PCA plot for RNA-Seq data shows the clustering of transcriptome by sample type (culture vs *in planta*) and time of sample collection (T1 vs. T2).

From the sequencing of *in planta* and *in vitro* cDNA libraries, approximately 164 million reads, comprising 95 million 76-bp-long reads, and 69 million 50-bp-long paired end reads were generated (Table 1). Approximately 87% of the reads from the *in vitro* libraries were mapped to the *S*. *sclerotiorum* genome. In contrast, 5.4–9.5% of the reads from the *in planta* libraries obtained from samples collected between 8 and 16 hpi, and approximately 38% of reads from the libraries obtained from samples collected between 24 and 48 hpi mapped to the reference *S*. *sclerotiorum* genome. On average, 0.6–1.5% of the reads mapped to *S*. *sclerotiorum* genome also mapped to *B*. *napus* genome, and these were excluded from the read counts for differential expression analysis. The percentage of reads from samples collected between 24 and 48 hpi that were mapped to the reference genome was 4 and 8 times larger than that of samples collected earlier for the susceptible and resistant lines, respectively. This difference was somewhat expected as it was apparent from the phenotypic observations that the fungal biomass at earlier stages was smaller compared to that at later stages (Fig 1a). Lower percentage of alignments at earlier phases of infection have also been observed in other pathogen-host interactions [34, 54, 55]. PCA results indicated that the differences in gene expression due to variability between replicates were small (9.15%, PC3, data not shown) compared to sample type (*in vitro* vs. *in planta*, PC1 63.1%) and time of sampling (T1 vs T2, PC2 15.4%) (Fig 2).

**Table 1:** Summary of the Illumina sequence reads obtained from *Brassica napus* plant inoculated with *S*. *sclerotiorum* and from mycelium of *S*. *sclerotiorum* isolate 1980 grown on potato dextrose agar.

	Time points	Total reads^1^	Reads mapped to reference *S*. *sclerotiorum* genome (%)
*In Vitro*	Culture	41665593	36323396 (87.18)
NEP32 (Susceptible)	8–16 hpi	37771674	2021544 (5.35)
	24–48 hpi	25360044	9642926 (38.02)
NEP63 (Resistant)	8–6 hpi	28670528	2728735 (9.52)
	24–48 hpi	31040632	11963775 (38.54)

^1^ Number of reads represent total of three biological replications

### Differential gene expression analysis

DGE analysis was conducted to detect *S*. *sclerotiorum* transcriptome changes during pathogenesis of canola. A total of 1301 and 1214 *S*. *sclerotiorum* genes were found to be differentially expressed at early (8–16 hpi) and late (24–48 hpi) stages of infection, respectively, during infection of the susceptible line (S1 File).

**Fig 3:**
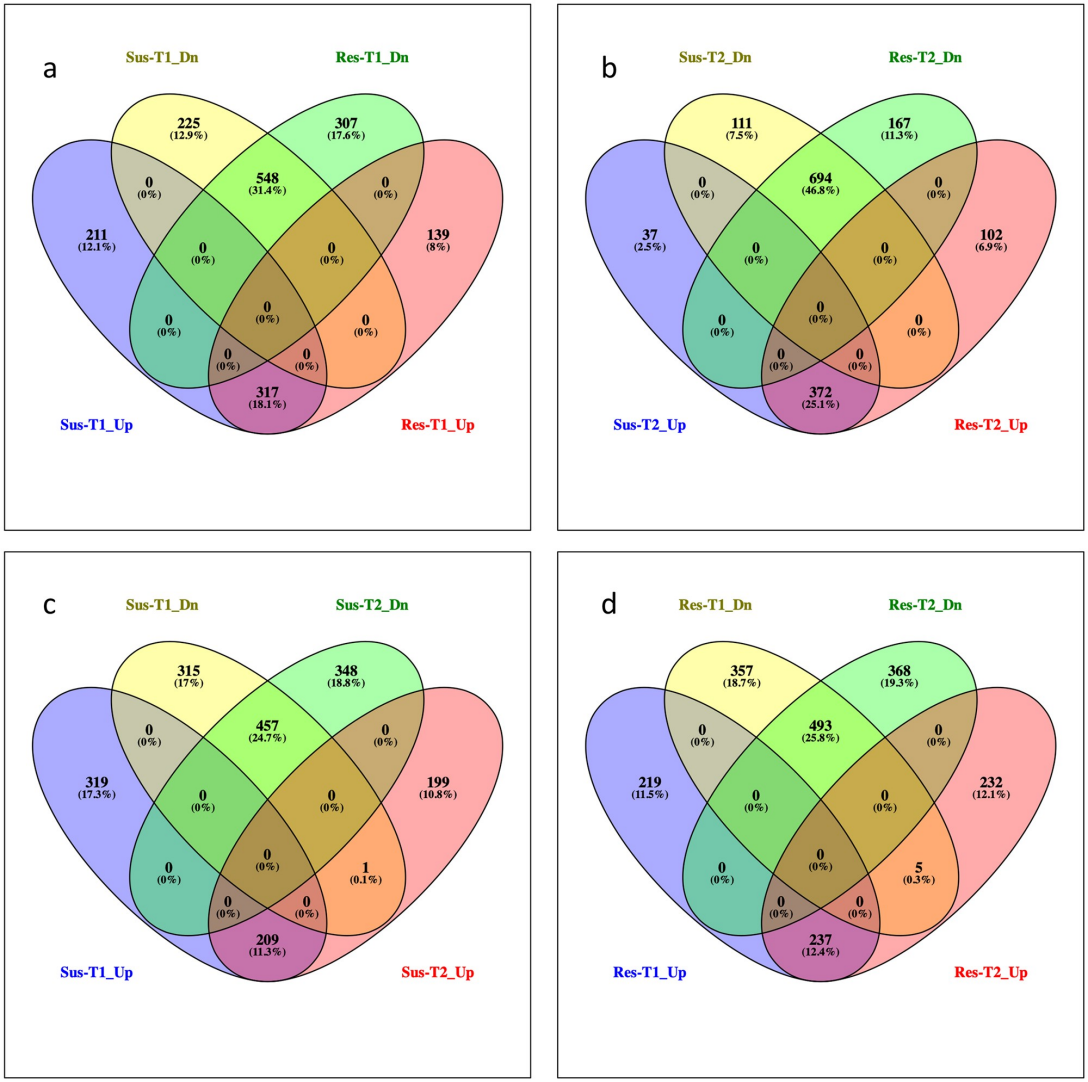
Venn diagrams showing differentially expressed *Sclerotinia sclerotiorum* genes during interaction with canola. Venn diagram shows the number of common and unique genes at (a) T1 and (b) T2, between up and down regulated gene sets of NEP32 and NEP63. (c) and (d) shows the comparison of up and down regulated genes at T1 and T2 gene sets of NEP32 and NEP63, respectively.

Of these, 528 and 773 genes were up- and down- regulated, respectively at T1, while 409 and 805 genes were up- and down- regulated, respectively at T2. When infecting the resistant line, 1311 and 1335 genes were differentially expressed at T1 and T2, respectively (Fig 3, S1 File). In this interaction, 456 and 474 genes were up-regulated, 855 and 861 genes were down-regulated at T1 and T2, respectively. At T1, 317 and 548 genes were common in up-regulated and down-regulated sets, respectively, between the susceptible and resistant interactions (Fig 3a). Similarly, there were 371 up-regulated and 694 down-regulated *S*. *sclerotiorum* genes that were common in both resistant and susceptible interactions at T2 (Fig 3b). When infecting the susceptible line, 209 up-regulated and 457 down-regulated genes were common between T1 and T2 (Fig 3c). The corresponding numbers when infecting the resistant line were 237 and 493 (Fig 3d). Regulation of an endo-glucanase gene, sscle_14g099920, shifted from significantly down-regulated at T1 to significant up-regulated at T2 in both susceptible and resistant interactions.

**Fig 4:**
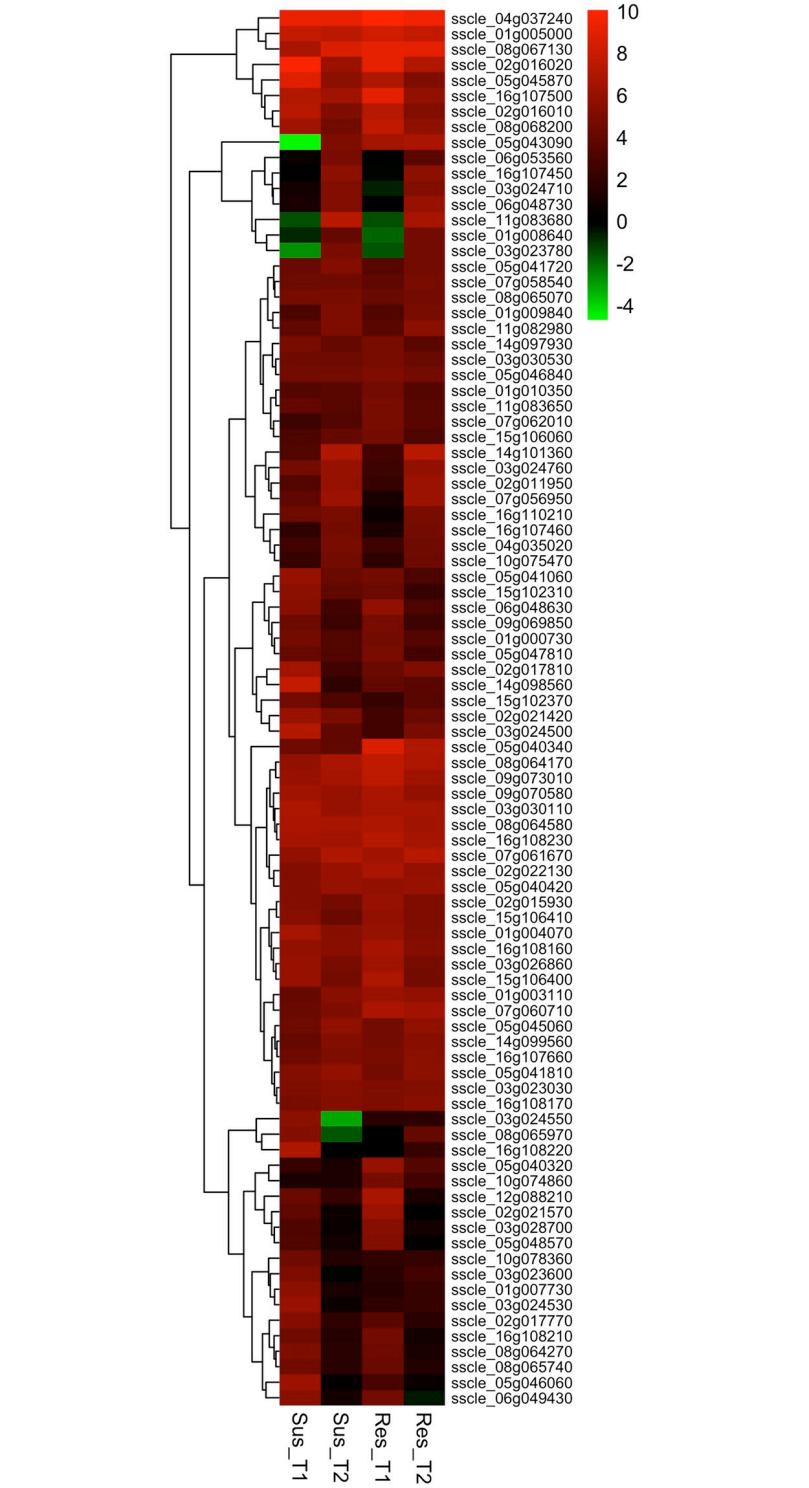
Heat maps showing expression patterns of the top 50 highly up-regulated *Sclerotinia sclerotiorum* genes from each treatment. Genes are grouped according to hierarchical clustering based on their expression patternsT1 and T2 represents earlier (8 and 16 hpi) and later (24 and 48 hpi) time points of interaction. The color gradient represents the log2 fold change in gene expression (up-regulation (red), down-regulation (green), and no change (black)) compared to *in vitro* control.

The fold-change in gene up-regulation ranged from 2 to 1053 (l2fc 1 to 10.04). In susceptible line, 12 and 8 genes were up-regulated over 100-fold at T1 and T2, respectively. In resistant line, 18 and 9 genes showed a similar 100-fold upregulation at T1 and T2, respectively. On average, approximately 50 genes were up-regulated by 25-fold (l2fc 4.64) or more in both susceptible and resistant lines at both time points. We made a comparison of the 50 most highly up-regulated genes from each of the interactions. Of these highly up-regulated genes, 22 were consistently up-regulated by 25-fold (l2fc 4.64) or more at both time points in both canola lines signifying their importance in pathogenesis (Fig 4 and S2 File). Five and 13 genes were common between susceptible and resistant lines at T1 and T2, respectively.

### GO categories and enrichment analysis

Functional characterization and gene functional enrichment analysis are powerful tools for analyzing DGE data to gain understanding of the important molecular pathways and functions underlying biological processes. *Sclerotinia sclerotiorum* DEGs were grouped according to their putative roles in biological processes (BP) and molecular functions (MF) as established by the Gene Ontology Consortium (http://www.geneontology.org/). *S*. *sclerotiorum* DEGs were assigned to the following GO classes: metabolic process, cellular process, cellular component organization, localization, biological regulation, response to stimulus, and signaling when interacting with susceptible and resistant lines at both time points.

GO enrichment analysis identified the key biological processes and molecular functions significantly enriched during pathogenesis. The up-regulated genes were significantly enriched with wide range of GO categories (S3 File). The significantly enriched categories included those involved in degradation of various cell wall components (including catabolism of cellulose (GO:0030245), xylan (GO:0045493), mannan (GO:0046355), pectin (GO:0045490)), peptidase activity (GO:0008233), oxidation-reduction processes (GO:0055114), response to xenobiotic stimulus (GO:0009410), fatty acid metabolic processes (GO:0006631), transmembrane transport (0055085), and binding (GO:0005488) activities.

GO enrichment analysis also provided insights into temporal aspects of pathogenesis. Genes involved in transcriptional reprogramming, e.g. gene expression (GO:0006396), ribosome biogenesis (GO:0042254), translation (GO:0006412), and cellular amino acid metabolic processes (GO:0006520) were enriched at the early infection stage, T1, in the up-regulated gene set indicating rapid transcriptional changes to adopt to pathogenic phase from *in vitro* phase. At the later infection stage, T2, many GO categories were overrepresented in the up-regulated set compared to the earlier stage. The significantly enriched up-regulated GO categories at later stage can be broadly grouped into enzymes involved in degradation of cell wall components (cell wall-degrading enzymes CWDE) and proteins, catabolism/detoxification of xenobiotic compounds and peroxisome associated pathways including peroxisome biogenesis, fatty acid catabolism and glyoxylate cycle.

### Differential gene expression data validation

**Fig 5:**
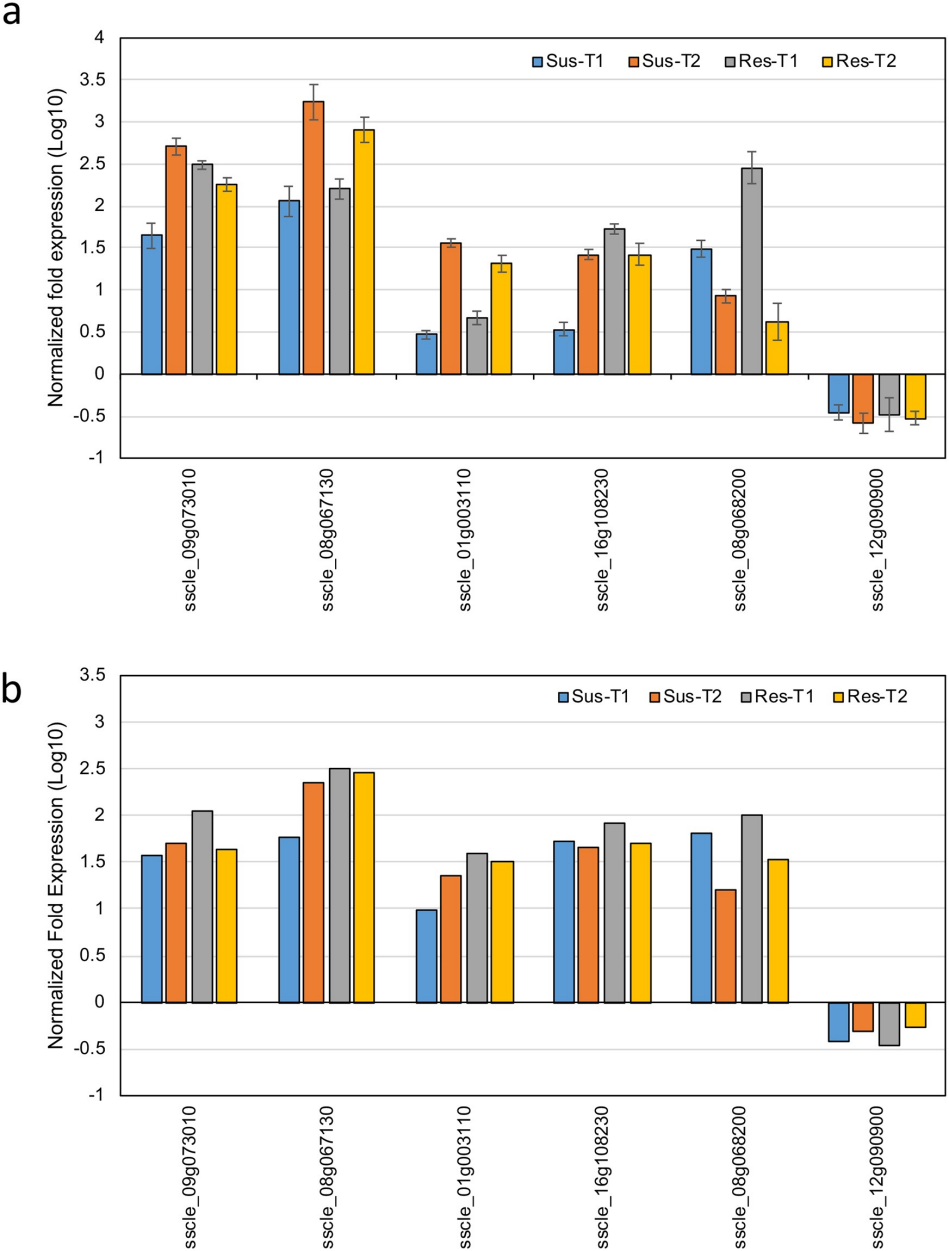
qRT-PCR validation of the relative expression levels of selected *S*. *sclerotiorum* differentially expressed genes. Expression profiles of six *S*. *sclerotiorum* genes as determined by a. qRT-PCR and b. RNA-Seq.

DGE data from RNA-seq analysis was validated by performing quantitative RT-PCR on five up-regulated DEGs and one down-regulated DEG. A list of the genes used for validation, their putative functions, and the primer sequences are presented in S4 File. The expression patterns of the six genes agreed with DGE data, thus validating the results of DGE analysis (Fig 5).

## Discussion

Both functional class enrichment analysis and the expression patterns of the highly up-regulated genes indicated that successful pathogenicity of *S*. *sclerotiorum* depends on cell wall degradation, detoxification and host defense evasion.

### CWDE and proteolytic enzymes

The importance of cell wall degrading activity was emphasized by the fact that the genes involved in this activity were both among the highly up-regulated and constituted many significantly enriched up-regulated GO categories. Approximately one third (29/90) of the highly up-regulated (top 50 genes up-regulated by 25-fold (l2fc 4.64) or more) *S*. *sclerotiorum* genes are involved in cell wall degrading enzymatic activities. Similarly, the significantly enriched CWDE GO categories included: hydrolases (GO:0016787) acting on: O-glucosyl bonds (GO:0004553), including cellulase (GO:0008810), glucosidase (GO:0015926), galactosidase (GO:0015925), polygalacturonase (GO:0004650), alpha-L-arabinofuranosidase (GO:0046556), beta-mannosidase (GO:0004567); ester bonds (GO:0016788), including aspartyl esterase (GO:0045330), lipase (GO:0016298), cutinase (GO:0050525), pectinesterase (GO: 0030599); peptidase (GO: 0008233), including serine-type endopeptidase (GO: 0004252), tripeptidyl-peptidase (GO:0008240) and serine-type carboxypeptidase (GO: 0004185) activities. The genome of *S*. *sclerotiorum* has a significantly large repository of both plant cell wall and fungal cell wall active enzymes [19]. Many of these enzymes were up-regulated in the current study (Table 2). Consistent with our findings, previous comparative transcriptome analyses [19, 28–30, 34–36] have reported that a large number of cell wall-degrading enzymes were up-regulated during pathogenesis, while at least one functional study demonstrated that disruption of the cell wall-degrading enzyme arabinofuranosidase/xylosidase (SS1G_02462) resulted in decreased virulence on canola [10].

**Table 2:** Changes in expression levels of *S*. *sclerotiorum* genes involved in degradation of plant cell wall components during interaction with susceptible (Sus) and resistant (Res) canola lines at early (T1, 8–16 hpi) and late (T2, 24–48 hpi).

gene_id	version1id	Sus-T1		Sus-T2		Res-T1		Res-T2		Activity
		l2fc	fdr	l2fc	fdr	l2fc	fdr	l2fc	fdr	
sscle_10g077670	SS1G_08493	0.89	0.788	-1.05	0.196	-0.87	0.366	-0.96	0.223	**Cellulose**
sscle_03g023660	SS1G_00891	1.66	0.383	1.67	0.402	1.73	0.373	1.23	0.556	
sscle_01g008530	SS1G_01485	-0.47	0.653	-1.00	0.230	-2.23	0.057	-1.50	0.065	
sscle_15g105560	SS1G_09365	0.59	0.670	-2.74	0.036	0.40	0.821	-2.42	0.040	
sscle_07g056950	SS1G_03387	3.84	0.145	6.23	0.032	1.12	0.816	6.22	0.030	
sscle_14g099920	SS1G_08837	-3.12	0.038	2.29	0.048	-3.59	0.025	2.48	0.032	
sscle_08g063000	SS1G_04945	3.37	0.058	1.95	0.260	2.97	0.084	1.80	0.293	
sscle_01g005720	SS1G_01828	0.52	0.571	2.16	0.008	0.92	0.269	2.50	0.003	
sscle_01g001690	SS1G_09821	-5.48	0.420	1.64	0.526	-5.48	0.370	-1.12	0.728	
sscle_04g039100	SS1G_03041	1.76	0.201	1.58	0.236	1.86	0.188	1.40	0.267	
sscle_03g028060	SS1G_00321	0.37	0.882	0.41	0.812	-0.80	0.715	0.84	0.571	
sscle_03g026840	SS1G_00471	-1.56	0.010	-1.19	0.028	-1.34	0.028	-1.36	0.013	
sscle_05g045320	SS1G_06037	-2.75	0.227	-1.04	0.546	-4.02	0.126	-0.38	0.855	
sscle_04g033820	SS1G_02334	2.78	0.007	4.24	0.000	1.88	0.049	4.30	0.000	
sscle_14g101360	SS1G_09020	3.33	0.143	6.97	0.013	2.82	0.270	7.29	0.010	
sscle_03g023650	SS1G_00892	2.80	0.007	4.36	0.000	2.62	0.011	4.37	0.000	
sscle_14g102070	SS1G_09118	2.08	0.386	-1.19	0.573	-4.65	0.251	-0.77	0.711	
sscle_04g033090	SS1G_02245	-3.23	0.007	-3.34	0.002	-4.05	0.006	-3.88	0.001	
sscle_10g080790	SS1G_13872	-11.04	0.061	-5.78	0.039	-7.68	0.085	-5.41	0.036	
sscle_04g035060	SS1G_02501	-2.53	0.017	-2.15	0.023	-3.92	0.004	-1.90	0.035	
sscle_14g100430	SS1G_08907	-1.66	0.003	-2.54	0.000	-1.98	0.001	-2.60	0.000	
sscle_11g082770	SS1G_07863	-2.22	0.328	-0.85	0.668	-3.56	0.189	-1.34	0.447	
sscle_08g064580	SS1G_05151	6.75	0.002	6.66	0.001	6.89	0.002	6.41	0.002	
sscle_08g064310	SS1G_05118	0.57	0.478	-0.64	0.438	0.03	0.978	-1.50	0.051	
sscle_01g007050	SS1G_01662	2.89	0.001	3.47	0.000	3.19	0.000	3.23	0.000	
sscle_15g105550	SS1G_09366	2.72	0.000	3.96	0.000	2.10	0.001	3.76	0.000	
sscle_05g043280	SS1G_06304	-4.06	0.037	-0.26	0.890	-3.96	0.056	0.13	0.949	
sscle_11g082920	SS1G_07847	-2.34	0.259	-0.44	0.804	0.43	0.875	0.49	0.771	
sscle_06g051500	SS1G_07146	1.56	0.653	3.24	0.222	-3.14	0.487	3.07	0.236	
sscle_03g030980	SS1G_13255	-3.78	0.018	-1.59	0.137	-2.69	0.063	-2.00	0.057	
sscle_08g066260	SS1G_05368	2.70	0.184	2.68	0.154	0.81	0.799	2.44	0.185	
sscle_06g053450	SS1G_12622	-1.19	0.239	1.34	0.163	-1.41	0.208	1.62	0.085	
sscle_06g051350	SS1G_07162	2.01	0.018	1.97	0.019	1.53	0.058	1.79	0.027	
sscle_14g102160	SS1G_09129	2.69	0.009	3.78	0.001	1.98	0.039	3.57	0.001	
sscle_02g018140	SS1G_04264	2.30	0.007	1.69	0.029	2.15	0.009	1.72	0.025	
sscle_03g022640	SS1G_01021	2.25	0.088	3.50	0.012	1.50	0.330	3.16	0.018	
sscle_16g108170	SS1G_10167	4.78	0.018	5.31	0.011	4.99	0.015	5.40	0.010	**Pectin**
sscle_09g070580	SS1G_10698	6.42	0.002	5.98	0.002	6.74	0.001	5.83	0.002	
sscle_05g046840	SS1G_05832	4.62	0.006	4.76	0.005	4.98	0.004	4.60	0.005	
sscle_04g035440	SS1G_02553	-2.04	0.363	2.58	0.064	-3.43	0.187	2.81	0.040	
sscle_02g018610	SS1G_04207	0.27	0.978	6.25	0.063	-4.95	0.702	6.39	0.054	
sscle_05g040500	SS1G_12057	0.41	0.762	0.31	0.953	-0.74	0.612	0.36	0.783	
sscle_07g055890	SS1G_03540	0.18	0.939	2.30	0.084	-2.82	0.190	1.82	0.162	
sscle_16g107500	SS1G_10071	7.06	0.002	6.39	0.003	9.02	0.001	5.80	0.005	
sscle_11g085640	SS1G_14449	1.42	0.064	2.36	0.005	1.72	0.030	2.56	0.003	
sscle_02g015930	SS1G_04551	5.34	0.006	4.68	0.010	6.08	0.003	5.07	0.005	
sscle_03g027970	SS1G_00332	3.67	0.002	3.75	0.001	3.46	0.003	3.56	0.002	
sscle_07g057800	SS1G_03286	4.10	0.076	4.10	0.082	4.12	0.074	3.93	0.087	
sscle_03g026860	SS1G_00468	6.05	0.000	4.90	0.000	6.32	0.000	4.83	0.000	
sscle_02g019490	SS1G_04095	-1.26	0.187	0.18	0.892	-1.06	0.280	0.67	0.507	
sscle_05g040420	SS1G_12048	5.26	0.001	6.03	0.000	5.82	0.001	6.08	0.000	
sscle_12g089930	SS1G_11992	3.16	0.099	4.27	0.012	4.23	0.015	4.27	0.010	
sscle_01g002020	SS1G_09857	-0.86	0.836	3.77	0.046	-1.30	0.725	3.98	0.033	
sscle_15g103150	SS1G_13501	0.99	0.287	3.20	0.002	-0.81	0.501	3.16	0.002	
sscle_02g016000	SS1G_04541	0.13	0.943	0.14	0.940	0.33	0.850	0.80	0.538	
sscle_10g075640	SS1G_08229	-6.87	0.013	-3.25	0.082	-4.81	0.036	-3.00	0.099	
sscle_06g052290	SS1G_07039	1.32	0.349	-0.04	0.982	0.64	0.727	0.95	0.455	
sscle_02g015920	SS1G_04552	3.23	0.039	1.38	0.379	3.70	0.024	1.24	0.437	
sscle_05g041650	SS1G_12191	1.27	0.370	3.85	0.016	1.05	0.483	3.97	0.012	**Xylan**
sscle_16g107660	SS1G_10092	4.37	0.000	5.07	0.000	4.89	0.000	5.54	0.000	
sscle_09g074790	SS1G_03618	-3.94	0.675	3.29	0.256	-3.94	0.619	2.28	0.460	
sscle_11g083680	SS1G_07749	-1.52	0.906	7.39	0.039	-1.52	0.879	6.63	0.052	
sscle_08g064500	SS1G_05140	-1.88	0.118	-1.16	0.287	-2.66	0.057	-1.31	0.218	
sscle_11g080920	SS1G_08104	2.29	0.076	3.93	0.004	4.20	0.004	2.84	0.019	
sscle_08g066710	SS1G_05434	0.32	0.882	2.92	0.028	0.10	0.968	2.87	0.027	
sscle_03g024710	SS1G_00746	0.88	0.758	5.18	0.016	-0.67	0.864	5.23	0.013	
sscle_05g045850	SS1G_05977	1.15	0.451	3.90	0.008	0.49	0.832	3.82	0.007	
sscle_10g075470	SS1G_08208	2.23	0.043	4.69	0.000	1.93	0.106	4.35	0.000	
sscle_10g074850	SS1G_08118	-1.88	0.072	0.04	0.972	-3.48	0.024	-0.14	0.918	
sscle_15g105540	SS1G_09367	-1.11	0.248	0.75	0.458	-1.80	0.101	0.57	0.588	
sscle_07g060690	SS1G_11535	-1.33	0.255	0.86	0.359	-1.52	0.200	0.77	0.412	
sscle_02g015110	SS1G_04662	-3.85	0.001	-2.62	0.002	-6.69	0.000	-2.53	0.002	
sscle_07g056960	SS1G_03386	0.22	0.935	3.28	0.019	-1.86	0.428	2.95	0.028	
sscle_11g082440	SS1G_07904	0.70	0.485	3.55	0.000	-2.29	0.084	3.52	0.000	
sscle_04g034810	SS1G_02462	2.71	0.022	4.10	0.002	1.12	0.387	4.29	0.002	
sscle_07g055410	SS1G_03602	2.13	0.085	3.65	0.006	1.76	0.161	3.50	0.007	
sscle_01g010480	SS1G_01216	-5.59	0.033	-3.31	0.067	-11.82	0.019	-3.27	0.059	**Arabinogalactans**
sscle_07g061080	SS1G_11585	0.64	0.830	3.64	0.034	-0.65	0.820	4.02	0.020	
sscle_04g035910	SS1G_02618	-2.48	0.008	0.05	0.962	-4.89	0.001	-0.01	0.993	
sscle_09g069470	SS1G_10842	3.20	0.001	3.54	0.000	1.65	0.045	3.31	0.001	
sscle_12g091670	SS1G_11763	-1.51	0.137	-0.18	0.974	-2.33	0.053	-0.35	0.713	
sscle_01g007810	SS1G_01572	-0.35	0.859	1.44	0.257	-3.06	0.147	1.07	0.412	
sscle_09g074570	SS1G_03647	1.51	0.126	2.96	0.008	0.61	0.612	2.69	0.012	
sscle_04g037140	SS1G_02781	1.57	0.078	1.42	0.111	1.23	0.174	1.35	0.122	
sscle_01g002110	SS1G_09866	0.29	0.925	2.49	0.188	-2.66	0.397	2.28	0.219	
sscle_12g090430	SS1G_11922	-5.34	0.569	3.63	0.186	-5.34	0.514	3.64	0.171	
sscle_01g010330	SS1G_01238	-0.43	0.834	-2.29	0.112	-0.51	0.790	-2.08	0.147	
sscle_04g035930	SS1G_02620	-1.36	0.358	0.95	0.452	-4.00	0.058	0.84	0.512	
sscle_09g069260	SS1G_10867	0.15	0.932	-1.92	0.117	1.30	0.344	-1.65	0.152	**Mannan**
sscle_02g016530	SS1G_04468	1.53	0.261	3.46	0.013	1.54	0.344	3.65	0.009	
sscle_02g011730	SS1G_12937	0.98	0.550	0.14	0.944	0.58	0.816	-0.08	0.968	
sscle_08g064250	SS1G_05110	-3.21	0.004	-2.91	0.001	-3.15	0.007	-2.94	0.001	
sscle_07g061030	SS1G_11579	-0.57	0.733	-0.67	0.654	-0.11	0.960	-1.99	0.132	
sscle_15g106590	SS1G_09229	-2.94	0.054	-0.38	0.815	-5.67	0.013	-0.40	0.809	
sscle_03g026560	SS1G_00505	-3.52	0.009	-3.19	0.003	-2.14	0.081	-2.13	0.016	
sscle_02g019080	SS1G_04148	-1.66	0.011	-0.54	0.365	-2.63	0.002	-0.73	0.202	
sscle_01g004220	SS1G_02022	-3.61	0.002	-1.18	0.147	-4.42	0.002	-1.30	0.105	
sscle_02g018660	SS1G_04200	-0.04	0.989	-2.10	0.233	-4.71	0.177	-0.84	0.656	
sscle_01g009600	SS1G_01334	-2.64	0.030	-1.83	0.045	-2.90	0.037	-1.24	0.151	

### Detoxification of xenobiotic compounds

Genes with known roles in detoxification and host defense evasion constituted the next group of highly up-regulated genes after the CWDEs (Table 3). These include: two laccases (sscle_03g023030, sscle_02g021570), nitrilase (sscle_16g108230), brassinin glycosyltransferase (sscle_01g003110, *SsBGT1*), two glutathione-S-transferases (GST), two cytochrome P450 monooxygenases, and three transporter genes, including two major facilitator superfamily transporters, three S-adenosyl methionine (SAM) dependent methyltransferases including a thiol methyltransferase.

**Table 3:** Changes in expression levels of *S*. *sclerotiorum* genes involved in detoxification of xenobiotic compounds and peroxisome associated pathways during interaction with susceptible (Sus) and resistant (Res) canola lines at early (T1, 8–16 hpi) and late (T2, 24–48 hpi).

gene_id	version 1 id	Sus-T1		Sus-T2		Res-T1		Res-T2		Activity/pathway
		l2fc	fdr	l2fc	fdr	l2fc	fdr	l2fc	fdr	
sscle_01g007350	SS1G_01627	1.00	0.572	-1.74	0.267	-0.75	0.714	-1.71	0.269	**Laccase**
sscle_03g023030	SS1G_00974	5.13	0.007	5.34	0.005	5.15	0.007	5.17	0.006	
sscle_02g018680	SS1G_04196	-1.96	0.121	-4.09	0.003	-2.52	0.085	-4.53	0.001	
sscle_08g064260	SS1G_05112	-1.26	0.435	-3.28	0.029	-5.94	0.038	-3.48	0.017	
sscle_12g090390	SS1G_11927	-3.80	0.067	-4.05	0.018	-2.53	0.171	-3.18	0.030	
sscle_02g021570	SS1G_13036	3.80	0.034	0.67	0.744	6.15	0.003	0.19	0.939	
sscle_13g092370	SS1G_06365	0.75	0.711	-1.91	0.341	-1.74	0.461	-0.21	0.936	
sscle_01g003110	SS1G_09997	4.10	0.001	5.42	0.000	6.13	0.000	5.82	0.000	**glucosyltransferase**
sscle_02g016980	SS1G_04416	0.30	0.709	1.22	0.057	1.34	0.060	1.28	0.043	
sscle_03g023640	SS1G_00894	0.90	0.292	-0.62	0.484	0.06	0.968	-0.34	0.727	
sscle_05g046370	SS1G_05901	-1.47	0.243	-1.26	0.249	-3.60	0.039	-1.30	0.215	
sscle_08g062730	SS1G_04910	-0.14	0.951	0.41	0.802	-0.19	0.937	0.52	0.733	
sscle_11g081870	SS1G_07979	-0.80	0.134	-0.31	0.584	-1.09	0.066	-0.42	0.423	
sscle_12g088170	SS1G_11129	0.80	0.210	0.69	0.279	0.34	0.662	0.64	0.316	
sscle_15g103340	SS1G_13524	-3.05	0.064	-4.36	0.012	-4.70	0.033	-4.34	0.010	
sscle_15g106380	SS1G_09252	-2.18	0.070	-2.86	0.008	-2.71	0.039	-2.54	0.010	
sscle_10g079920	SS1G_13754	-4.52	0.002	-0.28	0.838	-4.38	0.002	0.40	0.761	**cyanide hydratase**
sscle_16g108230	SS1G_10174	6.55	0.000	6.41	0.000	7.17	0.000	6.51	0.000	
sscle_01g007130	SS1G_01652	-3.22	0.021	-0.84	0.402	-3.23	0.053	-1.03	0.286	
sscle_07g060330	SS1G_11485	-0.17	0.859	1.02	0.130	-0.68	0.391	0.98	0.142	
sscle_01g008520	SS1G_01487	-2.53	0.020	-2.66	0.006	-5.99	0.002	-3.52	0.001	
sscle_15g104110	SS1G_09546	-1.08	0.132	-0.49	0.455	-1.70	0.050	-0.69	0.256	
sscle_16g108980	SS1G_10281	-0.42	0.519	-0.54	0.339	-1.24	0.070	-0.17	0.818	
sscle_03g030680	SS1G_13215	-0.73	0.628	0.25	0.863	-0.49	0.772	0.06	0.973	
sscle_13g096730	SS1G_14415	0.43	0.823	0.49	0.743	-1.01	0.580	0.33	0.837	
sscle_01g005000	SS1G_01918	7.74	0.000	7.51	0.000	8.20	0.000	7.70	0.000	**glutathione-S-transferase**
sscle_16g107740	SS1G_10108	-3.67	0.016	-4.00	0.004	-3.39	0.029	-4.99	0.001	
sscle_10g075490	SS1G_08210	-1.61	0.069	-0.59	0.471	-2.60	0.021	-0.72	0.358	
sscle_08g062750	SS1G_04914	-0.91	0.419	-0.99	0.311	-1.24	0.305	-1.39	0.125	
sscle_06g051110	SS1G_07195	-0.49	0.398	0.66	0.237	-0.81	0.167	0.79	0.142	
sscle_13g096550	SS1G_14440	0.25	0.892	0.72	0.588	1.29	0.297	1.17	0.317	
sscle_15g104750	SS1G_09479	-0.33	0.830	0.38	0.749	-0.60	0.661	0.73	0.480	
sscle_10g075830	SS1G_08258	-0.87	0.457	0.09	0.954	1.71	0.092	0.20	0.892	
sscle_06g053300	SS1G_12640	1.97	0.006	2.36	0.001	1.83	0.009	2.24	0.001	
sscle_08g067590	SS1G_05554	-1.46	0.102	-0.59	0.498	-1.69	0.082	-0.34	0.729	
sscle_01g008020	SS1G_01545	-2.18	0.038	-1.68	0.048	-1.99	0.068	-1.70	0.036	
sscle_13g094100	SS1G_06623	-0.19	0.876	-0.48	0.589	-0.66	0.485	-0.29	0.771	
sscle_01g004960	SS1G_01922	-0.06	0.962	-0.31	0.757	0.24	0.835	-0.01	0.990	
sscle_11g083650	SS1G_07752	3.97	0.001	3.54	0.001	4.93	0.000	3.56	0.001	
sscle_01g004270	SS1G_02014	1.66	0.043	0.03	0.979	0.51	0.600	0.06	0.961	**Chitin Binding or LysM effector**
sscle_02g014090	SS1G_04786	-0.48	0.735	-0.70	0.534	-0.70	0.604	-0.30	0.822	
sscle_03g024480	SS1G_00772	2.35	0.012	1.53	0.067	2.35	0.015	1.11	0.174	
sscle_03g025390	SS1G_00642	-3.63	0.037	-3.63	0.011	-2.16	0.223	-4.09	0.005	
sscle_05g042890	SS1G_12336	3.25	0.001	2.49	0.004	2.89	0.003	2.57	0.003	
sscle_06g054140	SS1G_12513	-8.68	0.010	-5.19	0.003	-4.41	0.030	-4.31	0.005	
sscle_06g054180	SS1G_12509	-10.20	0.032	-10.20	0.009	-10.20	0.033	-10.20	0.007	
sscle_08g068200	SS1G_14184	6.39	0.018	4.59	0.049	7.56	0.006	5.74	0.015	
sscle_15g105380	SS1G_09392	-3.52	0.028	-1.60	0.192	-4.45	0.028	-1.67	0.158	
sscle_15g107050	SS1G_09169	-1.39	0.605	-4.37	0.071	-0.52	0.902	-3.87	0.072	
sscle_07g062010	SS1G_11700	2.54	0.020	3.40	0.002	4.87	0.000	3.59	0.001	
sscle_08g066850	SS1G_05454	-2.69	0.077	-2.89	0.031	-3.45	0.056	-2.59	0.033	
sscle_08g066840	SS1G_05453	-2.36	0.282	-1.11	0.533	-7.05	0.067	-2.04	0.215	
sscle_05g041720	SS1G_12200	4.27	0.005	5.19	0.001	3.51	0.018	4.62	0.002	
sscle_03g027760	SS1G_00355	2.33	0.013	2.99	0.003	1.83	0.041	2.89	0.003	**nitronate monooxygenase**
sscle_12g087340	SS1G_11235	-1.62	0.374	1.97	0.222	-3.25	0.157	2.04	0.198	
sscle_11g085500	SS1G_14466	1.85	0.064	2.00	0.049	1.66	0.095	2.00	0.045	
sscle_09g069190	SS1G_10881	0.57	0.702	0.17	0.914	-1.42	0.384	0.20	0.897	
sscle_04g038190	SS1G_02923	0.03	0.976	2.06	0.007	-1.13	0.189	2.19	0.005	**fatty acid beta-oxidation**
sscle_16g108660	SS1G_10238	4.00	0.004	3.30	0.008	3.58	0.006	3.21	0.009	
sscle_03g028710	SS1G_00237	-0.31	0.727	1.42	0.045	0.05	0.970	1.65	0.020	
sscle_07g059730	SS1G_11414	0.81	0.238	2.21	0.004	1.24	0.092	2.26	0.003	
sscle_12g090420	SS1G_11923	-0.37	0.773	1.59	0.101	0.13	0.943	1.56	0.101	
sscle_09g069100	SS1G_10890	-0.38	0.793	1.35	0.231	-1.93	0.198	1.36	0.217	
sscle_10g076540	SS1G_08354	0.62	0.573	1.61	0.098	-0.38	0.785	1.97	0.042	
sscle_14g099760	SS1G_08821	0.64	0.479	0.83	0.363	0.23	0.835	1.02	0.246	
sscle_02g018210	SS1G_04249	1.89	0.276	1.27	0.406	1.29	0.459	2.42	0.069	
sscle_05g041330	SS1G_12152	-0.16	0.925	-0.53	0.690	0.29	0.840	-0.54	0.690	
sscle_11g086520	SS1G_14001	0.69	0.554	0.48	0.707	0.32	0.820	0.41	0.764	
sscle_09g069850	SS1G_10796	4.43	0.042	2.48	0.220	4.78	0.035	2.54	0.198	**oxalic acid**
sscle_14g099710	SS1G_08814	0.20	0.862	0.91	0.231	1.01	0.239	1.13	0.121	
sscle_10g075560	SS1G_08218	2.18	0.010	1.87	0.020	2.04	0.013	2.12	0.010	
sscle_08g062640	SS1G_04900	0.29	0.601	1.20	0.017	0.40	0.476	1.35	0.008	**glyoxylate cycle**
sscle_08g063200	SS1G_04975	3.50	0.001	4.20	0.000	3.72	0.001	4.39	0.000	
sscle_08g067810	SS1G_05583	1.29	0.047	2.78	0.001	2.06	0.005	2.92	0.000	
sscle_04g038190	SS1G_02923	0.03	0.976	2.06	0.007	-1.13	0.189	2.19	0.005	**Acetyl-CoA acetyl transferase**
sscle_09g069100	SS1G_10890	-0.38	0.793	1.35	0.231	-1.93	0.198	1.36	0.217	
sscle_10g075460	SS1G_08207	3.01	0.001	3.07	0.001	2.47	0.003	3.13	0.000	
sscle_05g041330	SS1G_12152	-0.16	0.925	-0.53	0.690	0.29	0.840	-0.54	0.690	

Laccases are multicopper oxidase enzymes that are known to detoxify phenolic compounds by oxidizing them [56]. Two of the seven predicted laccase genes in the *S*. *sclerotiorum* genome were up-regulated. Sscle_02g021570 (sslacc6) was up-regulated only at T1 (15–70 fold-change, l2fc 3.80–6.15). In contrast, Sslacc2 (sscle_03g023030) was consistently up-regulated (35–40 fold-change, l2fc 5.13–5.34) at both time points (Table 3). A similar expression pattern was observed in soybean–*S*. *sclerotiorum* interaction by Westrick et al. [34]. In *B*. *cinerea*, laccase gene BcLCC2, along with BcAtrB, an ABC transporter, was required for detoxification of the antifungal phenolic antibiotic 2,4-diacetylphloroglucinol [57]. Significantly enriched GO category xenobiotic metabolic processes (GO:0006805) also represented a catechol 1,2-dioxygenase gene, sscle_04g037100, involved in detoxification of host phenolic compounds. Catechol dioxygenases are induced in response to the phenolics produced by host plants [58]. The catechol dioxygenase gene CCHD1 was induced by maize phenolics [59], and was shown to be a virulence factor in the spruce pathogen *Endoconidiophora polonica* [60].

In addition to phenolic compounds, *Brassica* spp. are known to produce a wide range of phytoalexins, plant secondary metabolites that are elicited by biotic/ abiotic stress [61]; and in response to fungal pathogen attacks [62–64]. Production of phytoalexins has been considered a resistance determinant in some host-pathogen interactions [65]. The metabolism of these strongly antifungal compounds by pathogenic fungi, both *in vitro* and *in planta*, to less toxic compounds has been well researched [61]. Detoxification of cruciferous phytoalexins by *Sclerotinia sclerotiorum* involves glucosylation [66], a mechanism unusual for plant pathogens. Through genome mining and transcriptional profiling, Sexton et al. [67] identified several candidate glucosyltransferases including a brassinin glucosyl transferase (SsBGT1, sscle_01g003110). Consistent with Sexton et al. [67], the SsBGT1 gene was 17–70 fold up-regulated (l2fc 4.10–6.13) in our study (Table 3). The increased activity of this gene has also been reported by other researchers during infection of canola and soybean [34, 35]. Brassicas also are known to produce phytoanticipins like cyanogenic glucosides and glucosinolates [61, 68] which are produced as part of normal plant metabolism and could have strong antimicrobial properties. Non-toxic glucosinolates, upon cellular injury are hydrolyzed to produce highly toxic isothiocyanates (ITC) and nitriles [68]. In phytopathogenic fungi, nitrilases or cyanide hydratases play a role in detoxifying HCN to the less fungitoxic formamide [69, 70], which some fungi can utilize as a nitrogen source [71, 72]. In our study, the cyanide hydratase gene sscle_16g108230 was up-regulated by 85–145 folds (l2fc 6.41–7.17) at both time points. Cyanide hydratases/nitrilases, detected in the secretomes of *B*. *cinerea* [73], were up-regulated during infection of brassica hosts by *A*. *brassicicola*, *L*. *maculans* and *S*. *sclerotiorum* [35, 72, 74]. In *L*. *maculans*, increase in cyanide hydratase gene expression was induced in the presence of potassium cyanide or derivatives of brassica glucosinolates [72]. In our study, another cyanate hydratase gene, sscle_07g060330, was up-regulated at late stage only (> 2-fold in susceptible (l2fc 1.02), ≈ 2-fold (l2fc 0.98) in resistant line). Late activation of this gene was also observed by Seifbarghi et al. [35].

Glutathione-S-transferases (GSTs), are well known for their detoxification activity of xenobiotics and endogenous toxic compounds in fungi by their conjugation to glutathione [75, 76]. A GST gene, sscle_01g005000 was highly up-regulated (182–294 fold, l2fc 7.51–8.20) in our study (Table 3). Similarly high levels of up-regulation of this gene were reported during infection of canola [35] and soybean [34]. Sscle_11g083650, another GST, a membrane-associated protein in eicosanoid and glutathione metabolism (MAPEG), was also up-regulated by 12–30-fold (l2fc 3.54–4.93) in our study. Another GST, sscle_06g053300 was also consistently upregulated (4–5-fold, l2fc 1.83–2.36) (Table 3). In *A*. *brassicicola*, an ITC-inducible MAPEG class GST, AbMAPEG1, was required for full virulence on *B*. *oleracea* [77]. Deletion of two other *A*. *brassicicola* ITC-inducible GSTs, AbGSOT1 and AbUre2pB1, resulted in both hyper-susceptibility to ITC as well as impairment in pathogenicity [77]. These observations emphasize the importance of cyanogenic compound detoxification during pathogenesis.

SAM-dependent methyltransferases catalyze the transfer of methyl groups from SAM to diverse range of substrates [78]. In our study, three SAM-dependent methyltransferases, sscle_09g073010 (68–192-fold, l2fc 6.08–7.59), sscle_15g106060 (8.5–27-fold, l2fc 3.09–4.74) and sscle_08g065070 (19–30-fold, l2fc 4.24–4.92), were highly up-regulated (S2 File). In wood degrading fungus *Phanerochaete chrysosporium*, SAM transferases were involved in detoxifying phenolics [79]. In Brassica and Arabidopsis, thiol methyltransferases are known to detoxify glucosinolates [80].

Lysine motif, LysM, secreted effectors are proteins [81, 82] that contribute to mask the presence of plant pathogenic fungi in plant tissues by binding to chitin on the fungal cell walls [83–85] and interfering in this way with the plant’s ability to detect it [86–88]. In our study, two chitin-binding domain protein genes, sscle_08g068200 (24–189-fold, l2fc 4.60–7.56) and sscle_05g041720 (11–36-fold, l2fc 3.51–5.19) were found to be highly up-regulated. In addition to these two highly up-regulated genes, two other genes with chitin binding domain (sscle_01g004270 and sscle_07g062010) and a LysM effector gene (sscle_03g024480) were also up-regulated (Table 3). Regulation of four of these six genes followed a similar pattern, with highest up-regulation at T1 and gradually decreasing at T2. Up-regulation of LysM and chitin binding domain genes also were reported in *S*. *sclerotiorum* interactions with *B*. *napus* [35] and *G*. *max* [34], respectively.

Oxidative burst, characterized by a rapid and transient accumulation of reactive oxygen species (ROS) is one of the first plant defense responses to pathogen invasion [89], creating oxidative stress conditions hostile for the pathogens. Thus, coping with ROS is essential for pathogen survival and successful infection of the host. Fungal pathogens have evolved various mechanisms for tolerating or scavenging ROS, including peroxidases, catalases, superoxide dismutases (sod), and NADPH oxidases (nox). In our study, three peroxidases, sscle_01g000730 (10–26-fold, l2fc 3.35–4.73), sscle_08g065740 (3–24-fold, l2fc 1.43–4.59), and sscle_04g035020 (6–29-fold, l2fc 2.58–4.86) were highly up-regulated (Table 3). Nitronate monooxygenases (NMOs), FMN enzymes are known to play important role in oxidative detoxification of nitroalkanes [90]. Recently, Marroquin-Guzman et al. [91] showed that NMOs are involved in reactive nitrogen species (RNS) stress tolerance and suppressing host immune responses by maintaining cell redox status. In our study, two NMO genes, sscle_03g027760 and sscle_11g085500, were consistently up-regulated throughout the course of infection in both susceptible and resistant lines. A third NMO gene, sscle_12g087340 was down-regulated at T1 and up-regulated at only T2. There is evidence suggesting a brief biotrophic phase of *S*. *sclerotiorum* during the infection process and thus we speculate that apart from nitro-oxidative stress protection, *S*. *sclerotiorum’s* nitronate monooxygenases might have a similar role in suppressing host defenses during its interaction with canola plants.

*S*. *sclerotiorum* is known to employ OA for suppressing host defenses by manipulating host-redox environment [13]. OA, by far has been the most studied *S*. *sclerotiorum* virulence factor to date. In addition to manipulating the host redox environment, OA also plays a key role in virulence by acting in multiple ways: induction of programmed cell death [14], calcium chelating, and mediating pH signaling [15, 16]. We examined the expression of genes involved in OA metabolism. The gene sscle_10g075560, an oxaloacetate acetylhydrolase (OAH, *Ssoah1*) that is a key enzyme responsible for OA biogenesis and accumulation [92], was consistently up-regulated during infection (Table 3). The oxalate decarboxylase (OxDC) gene sscle_09g069850 (*Ss-odc2*), was also up-regulated in concert with *Ssoah1*. OxDC genes play an important role by preventing OA from being accumulated in fungal cell and thus protect the pathogen from its detrimental effects [93]. A similar expression pattern for *Ssoah1* and *Ss-odc2* was observed during infection of *B*. *napus* and *P*. *vulgaris*, respectively [33, 35], but not on *G*. *max* [34]. However, another OxDc gene, *Ss-odc1* (sscle_14g099710) was not differentially expressed (1–2 -fold change, l2fc 0.20–1.13). GO terms involved in production of OA precursors and oxidation- reduction process (GO:0055114) were also found to be significantly enriched in the DE gene sets.

### Peroxisome associated pathways

In addition to the above two broad classes, genes associated with peroxisomal pathways were consistently found to be significantly enriched in the up-regulated genes. The important GO categories significantly enriched/overrepresented in this broad group are: peroxisome organization (GO:0007031) including protein targeting (GO:0006625) and protein import (GO:0016558) into peroxisome matrix, fatty acid β-oxidation (GO:0006635) including and glyoxylate cycle (GO:0006097) (S3 File). Peroxisome-related metabolic functions are shown to be essential for pathogenic development of several plant pathogenic fungi [94]. Peroxisome biogenesis proteins, known as peroxins or PEX genes are involved in peroxisome biogenesis. PEX genes have been shown to be essential for pathogenicity/ virulence in the fungal pathogens *M*. *oryzae* [95–97], *C*. *orbiculare* [98–100] and *A*. *alternata* [101]. Up-regulation of large number of PEX genes in our study suggests a possible important role of these genes for *S*. *sclerotiorum* pathogenicity/ virulence.

Fatty acid β-oxidation is a lipid metabolic pathway for degrading long chain fatty acids for nutrient and energy generation [102, 103]. This is an enzyme mediated, four-step process pathway which results in acetyl-CoA, which can be fed into glyoxylate cycle or transported to mitochondria for energy generation through citric acid cycle. The carnitine acetyl transferase gene *pth2*, an appressorium-associated gene which catalyzes the transportation of acetyl-CoA is required for rice infection by *M*. *oryzae* [104]. *Ss-pth2*, a *S*. *sclerotiorum* ortholog of the *pth2* gene was found to be essential for host colonization [105], suggesting an essential role for peroxisomal pathways for host colonization and disease development. This gene (sscle_03g031670) was up-regulated during early and late stages of infection in both susceptible and resistant interactions in this study.

The glyoxylate cycle is important for gluconeogenesis, generation of glucose under nutrient scarce condition by assimilating the Acetyl-CoA generated via fatty acid β-oxidation. Isocitrate lyase (*ICL1*) and Maleate synthetase (*MSL1*) are two important enzymes of glyoxylate cycle [102]. *ICL1* was found to be essential for the pathogenicity of another canola pathogen *L*. *maculans* [106]; and was shown to be important for full virulence of *M*. *oryzae* [107] and *C*. *orbiculare* [108]. *Sclerotinia sclerotiorum* malate synthase gene (*mls1*) was shown be conditionally essential for fatty acid metabolism and pathogenicity on tomato [109].

### Secreted effectors

**Fig 6:**
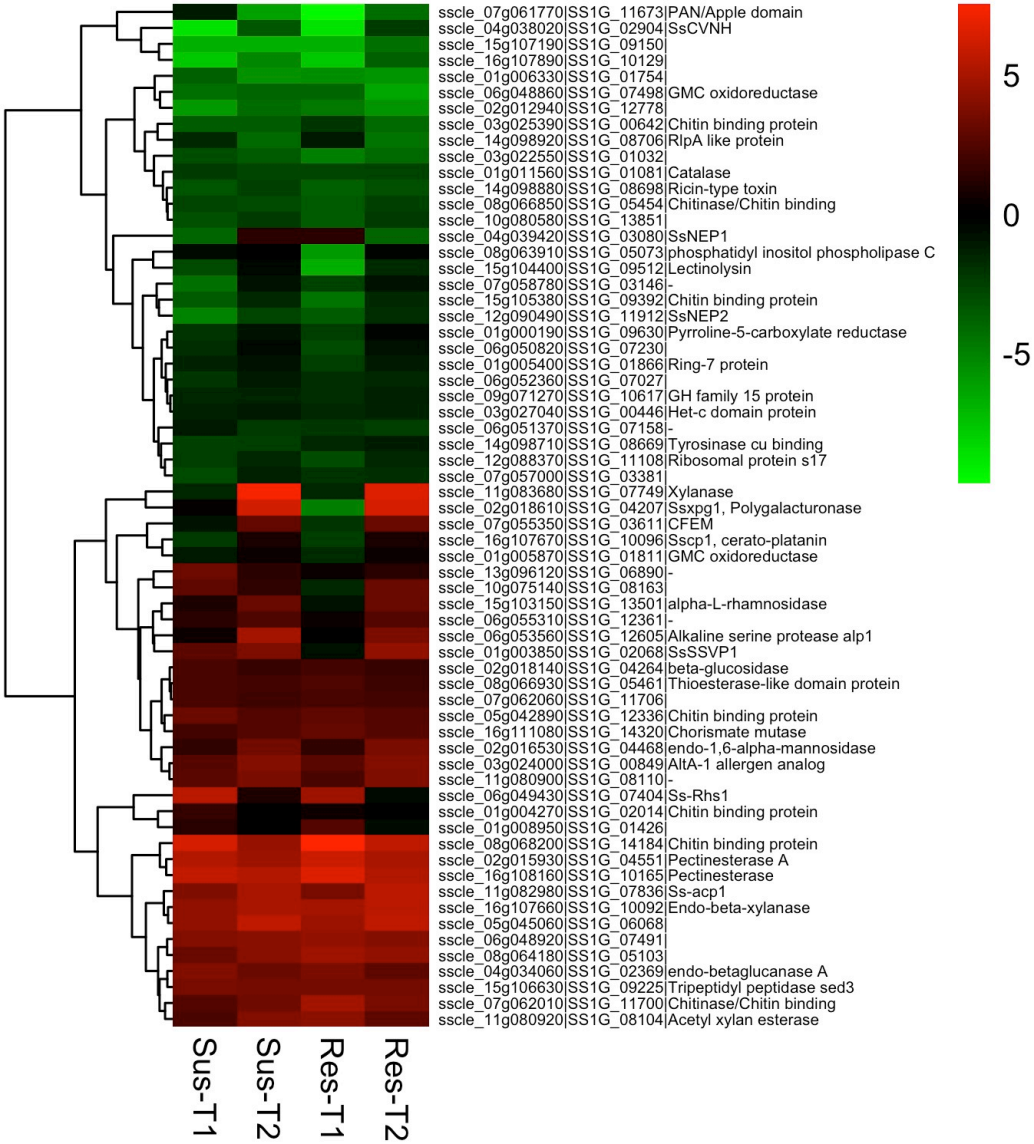
Heat maps showing expression patterns of *Sclerotinia sclerotiorum* effector genes. Genes are grouped according to hierarchical clustering based on their expression patterns. T1 and T2 represents earlier (8 and 16 hpi) and later (24 and 48 hpi) time points of interaction. The color gradient represents the log2 fold change in gene expression (up-regulation (red), down-regulation (green), and no change (black)) compared to *in vitro* control.

Secreted effector proteins play a key role in pathogenesis. Putative effector candidate genes were identified in the *S*. *sclerotiorum* genome based on bio-informatic analysis [31, 110]. We compared the expression of these *S*. *sclerotiorum* putative effector genes to determine specific temporal changes in their regulation. In total, 64 putative effector candidates were differentially regulated, of which 37 genes were up-regulated in at least one time point (Fig 6). The majority of these genes were consistently up-regulated in both susceptible and resistant lines at both time points. The up-regulated effector genes were mostly involved in CWDE activity, a non-aspartyl acid protease (*acp1*, sscle_11g082980), Rhs repeat containing protein (*Ss-rhs1*, sscle_06g049430), chorismate mutase (*SsCm1*, sscle_16g111080), and two chitin binding domain proteins (sscle_08g068200, sscle_11g082980). The gene *acp1* is induced in presence of cell walls and its expression is regulated by carbon, nitrogen starvation and pH [111]. Up-regulation of this gene increased from 12.5 -fold (l2fc 3.64) at T1 to 48 -fold (l2fc 5.58) at T2. A similar expression pattern was observed in *S*. *sclerotiorum* interactions with *P*. *vulgaris*, *B*. *napus* and *G*. *max* [33–35]. *Ss-Rhs1* was shown to be important for sclerotial development and initial infection on *B*. *napus* and Arabidopsis [22]. This gene was highly expressed during initial stages of sclerotial development and hyphal infection [22]. *Ss-Rhs1* was found to be highly up-regulated during the early infection stage (24 hpi) compared to the late infection (48–96 hpi) stage on *G*. *max* [34], implying a possibly important role during early infection process. Similarly, we also observed 26–48 -fold up-regulation (l2fc 4.72–5.56) of *Ss-Rhs1* only at T1 in both susceptible and resistant lines, respectively. In contrast, the gene *SsCm1* was up-regulated by 4–8.5 -fold (l2fc 2.02–3.08) in our study, which is similar to previous reports of high levels of expression in interactions with *B*. *napus* [24], and *G*. *max* [34] during early stages of infection. The biotrophic pathogen *Ustilago maydis* chorismate mutase gene *cmu1*, was shown to decrease SA levels in infected host tissue during infection of *Zea mays*, contributing to the virulence [112]. We speculate that *SsCm1* is likely involved in host manipulation like the *cmu1* in *U*. *maydis*–*Z*. *mays* interaction by interfering with SA signaling in *B*. *napus*. The role of chitin-binding proteins and LysM effectors in avoiding host recognition was discussed earlier. However, a few effector candidates (11) exhibited a shift in their regulation from down-regulation at T1 to up-regulation at T2. Interestingly four of these genes coding for a cerato-platanin (*SsCP1*, sscle_16g107670), CFEM domain containing (sscle_07g055350), a small secreted virulence-related protein *SsSSVP1* (sscle_01g003850, in resistant), and a necrosis and ethylene-inducing peptides *SsNep1* (sscle_04g039420, in susceptible) are known to function as necrotrophic effectors. Cerato-platanins are fungal specific, small secreted cysteine rich proteins known to function both as elicitors of plant defenses and as well as effectors contributing to virulence [113] by inducing localized necrosis of host tissue. In *S*. *sclerotiorum*, SsCP1 was shown to contribute to its virulence by directly interacting with pathogenesis-related protein PR1 [27]. In our study *Sscp1* was down-regulated at T1 by 4.9 -fold (l2fc -2.39) and up-regulated 1.8 -fold (l2fc 0.85) by T2. The CFEM, conserved fungal extracellular membrane proteins domain is a fungal specific domain containing eight conserved cysteine residues [114, 115] and are proposed to have role in fungal pathogenesis. The CFEM protein, *pth11* in *M*. *oryzae* is essential for appressoria development and pathogenesis [116, 117]. Kou et al. [117] showed that deletion of *pth11* results in disruption of redox homeostasis and thus affects appressorium formation during pathogenesis. In *B*. *cinerea*, a closely related broad-host-range necrotroph, *BcCFEM1*, a CFEM containing gene, plays a key role in stress resistance and virulence [118]. *BcCFEM1* also has a potential elicitor role. Gene Sscle_07g055350 was 4.4 -fold down-regulated (l2fc -2.13) at T1, followed by 9 -fold upregulation (l2fc 3.18) at T2. A similar expression pattern (up-regulation only at late infection) was observed by Seifbarghi et al [35] and Guyon et al. [110] during infection of canola and soybean [34] by *S*. *sclerotiorum* and by Thatcher et al. [119] during *Fusarium oxysporum* and *Medicago truncatula* interaction. *SsSSVP1*, another characterized *S*. *sclerotiorum* virulence factor, is a small cysteine rich secreted protein essential for full virulence. *SsSSVP1* interferes with the host mitochondrial respiratory pathway by interacting with the QCR8 subunit of cytochrome b-c1 complex, resulting in plant cell death [26]. Consistent with our study, expression of *SsSSVP1* was not detected until late infection stage (96 hpi) in *B*. *napus* [35]. *SsNep1* and *SsNep2* are shown to induce necrosis in host plants [25]. In this study, *SsNep1* was not up-regulated until T2 (2.5 -fold up-regulation, l2fc 1.35), with gradual increase from early infection (15 -fold down-regulation, l2fc -3.89). A similar expression pattern was also observed for *SsNep2* (-33 –-3 -fold change (l2fc -5.07 –-1.76) from T1 –T2). These two genes were induced mid—late stages of infection in *B*. *napus* and *G*. *max* [34, 35].

In contrast to the widely accepted necrotrophic nature of *S*. *sclerotiorum*, recent molecular and cytological evidences suggest a two-phase model involving a brief biotrophic or basic compatibility phase characterized by host suppression and subverting of host defenses following by necrotrophic phase [38]. The temporal differential expression patterns of the effector candidates in our study supports the two-phase infection model [38–40] proposed for *S*. *sclerotiorum*. Biotrophic effectors like chorismate mutase (*SsCm1*), chitin binding proteins, LysM, and genes involved in ROS and RNS scavenging are up-regulated at early infection phase. Effectors known to induce necrosis like *SsCp1*, *SsSSVp1*, *SsNep1*, *SsNep2* were either not up-regulated until late infection and/or down-regulated at early stages of infection. A similar trend in expression of necrotrophic effectors was observed by Westrick et al in *S*. *sclerotiorum—G*. *max* interaction [34]. Two characterized effector genes *SsCVNH* [19] and *SsITL* [23] were significantly down-regulated at both time points, whereas another characterized effector gene, *Ssggt2* (sscle_09g068730), encoding γ-glutamyl transpeptidase was consistently up-regulated (2–3 -fold, l2fc 1.12–1.65) at both time points.

## Conclusions

This is the first study that examine global transcriptional changes in *S*. *sclerotiorum* during infection of canola plants differing in their susceptibility to the pathogen. The findings from this study emphasize the role of peroxisome related pathways, in addition to the cell wall degradation and detoxification of host metabolites as the key mechanisms underlying pathogenesis of *S*. *sclerotiorum* on canola. Further, temporal changes in expression pattern of several functional classes of genes, like expression of genes involved in avoiding host recognition or suppressing host defenses at early infection stage (Chitin binding domains, LysM effectors, ROS scavenging) and late onset of expression of necrosis inducing effectors (cerato-platanin, *SsSSVP1*, CFEM domain, *SsNep1* and *SsNep2* genes etc.) provided support for the proposed two-phase infection strategy involving a brief biotrophic phase during early infection. Functional analysis of these genes would provide further insight on the events that lead to disease development and colonization of plant tissues.

## Supporting information

S1 File List of differentially expressed *S*. *sclerotiorum* genes during interaction with susceptible [32] and resistant [63] canola lines at early (T1, 8–16 hpi) and late (T2, 24–48 hpi). (XLSX)

S2 File Changes in expression levels of the top 50 highly upregulated S. sclerotiorum genes during infection of susceptible [32] and resistant [63] canola lines at early (T1, 8–16 hpi) and late (T2, 24–48 hpi). (XLSX)

S3 File Enrichment analysis of *S*. *sclerotiorum* up-regulated genes during interaction with susceptible [32] and resistant [63] canola lines at early (T1, 8–16 hpi) and late (T2, 24–48 hpi). (XLSX)

S4 File List of selected *Sclerotinia sclerotiorum* genes, their putative functions, primers, and sequences used for quantitative RT-PCR. (XLSX)
